# 5-*epi*-Sinuleptolide from Soft Corals of the Genus *Sinularia* Exerts Cytotoxic Effects on Pancreatic Cancer Cell Lines via the Inhibition of JAK2/STAT3, AKT, and ERK Activity

**DOI:** 10.3390/molecules26226932

**Published:** 2021-11-17

**Authors:** Wan-Chi Tsai, Wen-Hung Wang, Bo-Cian Huang, Chiung-Yao Huang, Jyh-Horng Sheu

**Affiliations:** 1 Department of Medical Laboratory Science and Biotechnology, College of Health Sciences, Kaohsiung Medical University, Kaohsiung 807, Taiwan; kan59101043@hotmail.com; 2 Department of Laboratory Medicine, Kaohsiung Medical University Hospital, Kaohsiung 807, Taiwan; 3 Department of Medical Research, Kaohsiung Medical University Hospital, Kaohsiung 807, Taiwan; 4 Research Center for Environmental Medicine, Kaohsiung Medical University, Kaohsiung 807, Taiwan; 5 Department of Otolaryngology, Sijhih Cathay General Hospital, New Taipei City 221, Taiwan; ent.taiwan@gmail.com; 6 Department of Otolaryngology, Cathay General Hospital, Taipei 110, Taiwan; 7 School of Medicine, Fu Jen Catholic University, New Taipei City 242, Taiwan; 8 Department of Marine Biotechnology and Resources, National Sun Yat-sen University, Kaohsiung 804, Taiwan; huangcy@mail.nsysu.edu.tw; 9 Frontier Center for Ocean Science and Technology, National Sun Yat-sen University, Kaohsiung 804, Taiwan; 10 Department of Medical Research, China Medical University Hospital, China Medical University, Taichung 404, Taiwan

**Keywords:** soft corals, 5-*epi*-sinuleptolide, pancreatic cancer, cytotoxicity, STAT3

## Abstract

Pancreatic ductal adenocarcinoma is one of the most lethal malignancies: more than half of patients are diagnosed with a metastatic disease, which is associated with a five-year survival rate of only 3%. 5-*epi*-Sinuleptolide, a norditerpene isolated from *Sinularia* sp., has been demonstrated to possess cytotoxic activity against cancer cells. However, the cytotoxicity against pancreatic cancer cells and the related mechanisms are unknown. The aim of this study was to evaluate the anti-pancreatic cancer potential of 5-*epi*-sinuleptolide and to elucidate the underlying mechanisms. The inhibitory effects of 5-*epi*-sinuleptolide treatment on the proliferation of pancreatic cancer cells were determined and the results showed that 5-*epi*-sinuleptolide treatment inhibited cell proliferation, induced apoptosis and G2/M cell cycle arrest, and suppressed the invasion of pancreatic cancer cells. The results of western blotting further revealed that 5-*epi*-sinuleptolide could inhibit JAK2/STAT3, AKT, and ERK phosphorylation, which may account for the diverse cytotoxic effects of 5-*epi*-sinuleptolide. Taken together, our present investigation unveils a new therapeutic and anti-metastatic potential of 5-*epi*-sinuleptolide for pancreatic cancer treatment.

## 1. Introduction

Pancreatic ductal adenocarcinoma (PDAC) represents one of the leading causes of cancer-related mortality in developed countries and is the most lethal malignant neoplasm worldwide [1]. The mortality rate of pancreatic cancer is almost identical to its incidence [2]. Pancreatic cancer presents a considerable diagnostic challenge, and the majority of cases are diagnosed during advanced stages, with either locally advanced or metastatic disease. The prognosis of patients with pancreatic cancer has improved to a minor extent over the past two decades [3]. Surgery represents the only chance of cure; however, fewer than 20% of patients can undergo surgery because the cancer usually spreads beyond the pancreas when it is diagnosed. For all stages combined, the five-year relative survival rate is 10% [4].

**Figure 1:**
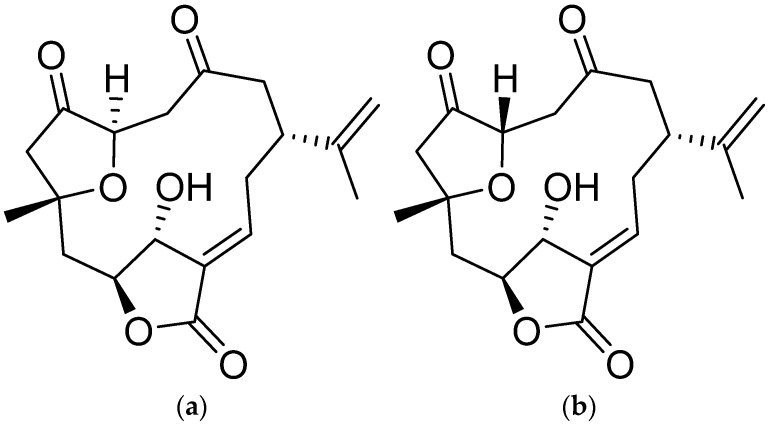
Chemical structure of sinuleptolide (**a**) and 5-*epi*-sinuleptolide (**b**).

Gemcitabine is a pyrimidine antimetabolite [5] that shows potent activity against various solid tumors and was approved by the US Food and Drug Administration in 1997 as a first-line treatment for pancreatic cancer [6]. However, the development of gemcitabine resistance in cancer cells leads to a low response to chemotherapy and remains a significant limitation in its use [7]. In 2011, FOLFIRINOX, a new treatment regimen, which combined 5-fluorouracil, leucovorin/folinic acid, oxaliplatin, and irinotecan, showed greater survival outcomes in patients with PDAC, compared to outcomes obtained using gemcitabine as a single agent, which led to the preferred option of using FOLFIRINOX as a treatment [2]. However, the high toxicity caused by this regimen limits its use [7]. The lack of effective anti-pancreatic cancer drugs prompted us to investigate bioactive compounds as alternative options for treating pancreatic cancer, especially natural products. The marine environment represents an exceptional reservoir comprising an enormous source of novel and biologically active compounds that are amenable to drug discovery [8]. Many marine natural products have been shown to possess significant pharmacological activities, in particular anticancer [9,10], anti-inflammatory [8], and antimicrobial properties [11], revealing the potential for the discovery and development of new medicines from marine environment. The soft corals of *Sinularia* genus are marine organisms well recognized for their capability to generate bioactive and structurally versatile natural products, among which two diastereomeric norcembranoids—sinuleptolide and 5-*epi*-sinuleptolide (Figure 1)—have been isolated from the soft corals *Sinularia leptoclados* [12] and *S. scabra* [13]. Later, both compounds were isolated again from the soft coral *S. lochmodes* of the same genus and the molecular structures with absolute configurations of both stereoisomers were finally fully established by chemical and NMR spectroscopic methods [14]. 5-*epi*-Sinuleptolide has been shown to display antibiotic activity by inhibition of anti-biofilm formation of bacteria [15], and both norcembranoids could also inhibit the production of nitric oxide and LPS-induced TNF-α in RAW 246.7 (murine macrophage) cells [16]. Further, both compounds were found to show cytotoxic activity against the growth of Hepa59T/VGH and KB cancer cell lines [12]. More detailed studies, including the mechanisms of cytotoxic effects of 5-*epi*-sinuleptolide toward human skin [17], and sinuleptolide against oral [18] cancer cells have been investigated; however, the cytotoxic effects of both compounds against the proliferation of pancreatic cancer cells have not yet been studied.

## 2. Results

### 2.1. 5-epi-Sinuleptolide Showed Selective Cytotoxicity against Pancreatic Cancer Cells

**Figure 2:**
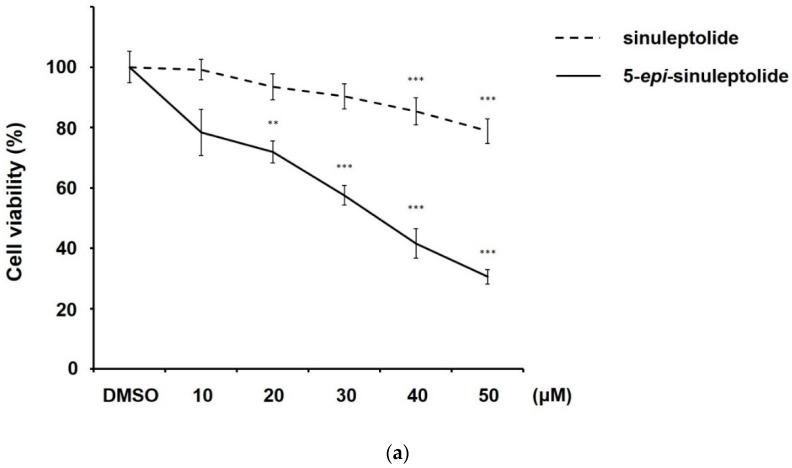

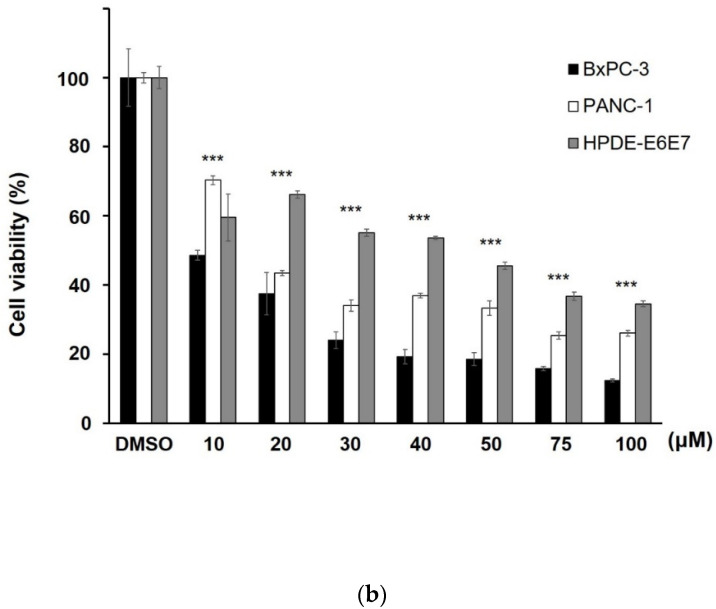
Selective cytotoxicity of 5-*epi*-sinuleptolide in pancreatic cancer cells. Cell viability was assessed by MTT assay after 24 h of treatment. Gemcitabine-sensitive BxPC-3 cells were incubated with different concentrations of sinuleptolide or 5-*epi*-sinuleptolide (**a**). The graph represents the mean of three experiments with the viability of DMSO-treated control normalized to 100% as the mean ± standard deviation. ** indicates *p* < 0.01, and *** *p* < 0.001 of sinuleptolide or 5-*epi*-sinuleptolide-treated BxPC-3 cells compared to DMSO-treated control. BxPC-3 with PANC-1 (gemcitabine-resistant), and HPDE-E6E7 (immortalized pancreatic cells) were exposed to 5-*epi*-sinuleptolide at indicated concentrations (**b**). The graph represents the mean of three experiments with the viability of DMSO-treated control normalized to 100% as the mean ± standard deviation. *** represents the *p*-value < 0.001 of 5-*epi*-sinuleptolide-treated BxPC-3, PANC-1, and HPDE-E6E7 cells compared to DMSO-treated control.

To evaluate the cytotoxicity of sinuleptolide and 5-*epi*-sinuleptolide, the gemcitabine-sensitive pancreatic cancer cell line BxPC-3 was treated with dimethyl sulfoxide (DMSO) or various concentrations of sinuleptolide or 5-*epi*-sinuleptolide for 24 h, and cell viability was analyzed via MTT assays (Figure 2a). Treatment with 5-*epi*-sinuleptolide resulted in a significant decrease in cell viability while sinuleptolide showed negligible cytotoxic effect. Hence, the 5-*epi*-sinuleptolide was chosen for the following study. To further examine whether 5-*epi*-sinuleptolide possessed a selective cytotoxicity, in addition to BxPC-3 cells, gemcitabine-resistant PANC-1 cells and HPDE-E6E7, the immortalized pancreatic duct epithelial cells were treated with DMSO or indicated concentrations of 5-*epi*-sinuleptolide for 24 h. The cytotoxic effects of 5-*epi*-sinuleptolide on pancreatic cancer cells were superior to those against pancreatic duct epithelial cells (Figure 2b). The half maximal inhibitory concentration of 5-*epi*-sinuleptolide associated with cytotoxicity in BxPC-3, PANC-1, and HPDE-E6E7 cells was 9.73, 17.57, and 44.54 μM, respectively. As BxPC-3 showed the highest sensitivity to 5-*epi*-sinuleptolide, it was used in the following experiments.

### 2.2. 5-epi-Sinuleptolide Inhibited Proliferation and Induced Apoptosis in BxPC-3 Cells

**Figure 3:**
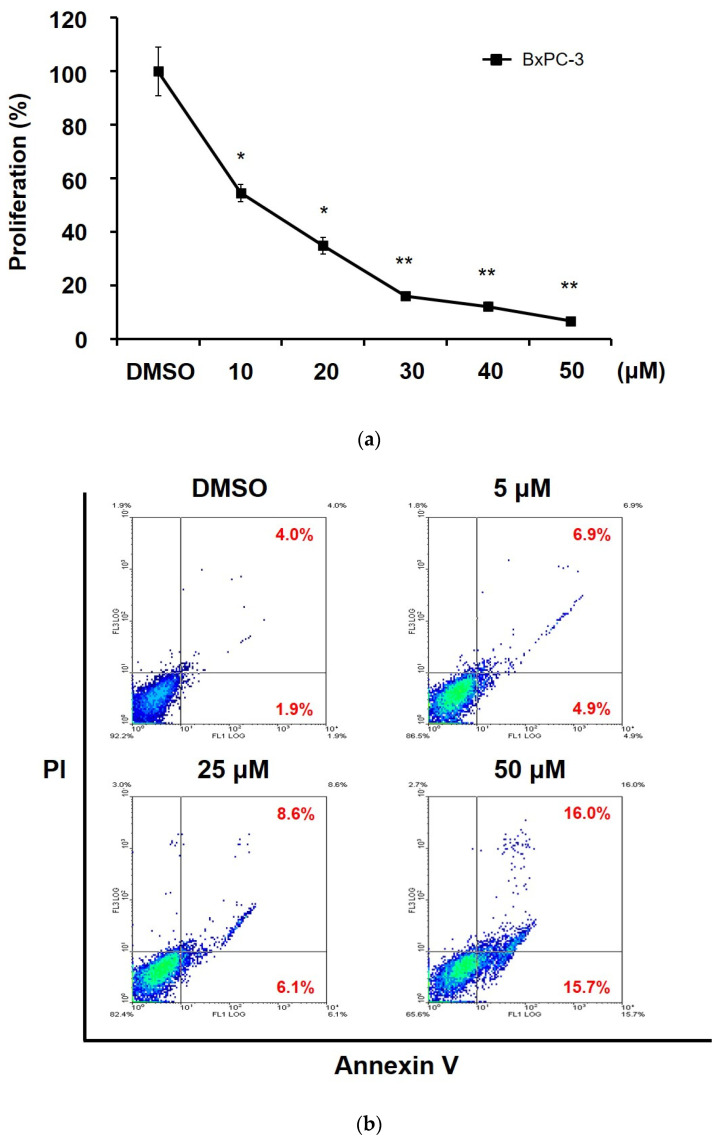

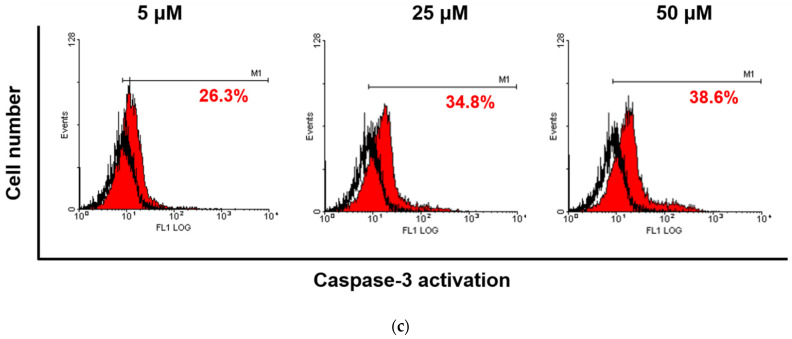
5-*epi*-Sinuleptolide inhibits BxPC-3 cells proliferation and induces apoptosis. BxPC-3 cells were exposed to 5-*epi*-sinuleptolide at the indicated concentrations, and cell proliferation rate was measured via the bromodeoxyuridine incorporation assay after 24 h. * indicates *p* < 0.05 vs. DMSO-treated control group, and ** indicates *p* < 0.01 (**a**). BxPC-3 cells treated with 5-*epi*-sinuleptolide for 24 h at the desired concentrations were stained with Annexin V-FITC and PI. The Annexin V-FITC signal is shown on the *X*-axis and the PI signal is shown on the *Y*-axis. Intact cells are located in the lower left quadrant, necrotic cells permeable to propidium iodide are in the upper left quadrant, and the apoptotic cells stained by annexin V and unstained by propidium iodide in the lower right quadrant. The bolded numbers represent the percentage of apoptosis or necrosis in the right lower or upper quadrant, respectively. (**b**). Caspase-3 activity of BxPC-3 cells treated with DMSO (open histogram) or 5-*epi*-sinuleptolide (red-filled histogram) for 24 h was measured by flow cytometry (**c**). FITC, fluorescein isothiocyanate; PI, propidium iodide; DMSO, dimethyl sulfoxide (**c**).

Next, we investigated whether the cytotoxicity of 5-*epi*-sinuleptolide was mediated by the suppression of cell proliferation and/or induction of apoptosis. BxPC-3 cells were labeled with bromodeoxyuridine (BrdU) for the quantification of cell proliferation after 24 h of treatment. Treatment with 5-*epi*-sinuleptolide at 10, 20, 30, 40, and 50 μM was associated with 54.6%, 34.9%, 16.1%, 12.2%, and 6.8% of cell proliferation, respectively, compared to that of DMSO-treated control cells (Figure 3a). Furthermore, cell death induced by 5-*epi*-sinuleptolide was evaluated via flow cytometry. The proportion of cells showing Annexin V-FITC+/PI- and those showing Annexin V-FITC+/PI+ were defined as apoptosis and necrosis, respectively. After 24 h of treatment with 5-*epi*-sinuleptolide, a dose-dependent increase in the right lower and upper quadrant were observed (Figure 3b). Treatment with 5, 25, and 50 μM of 5-*epi*-sinuleptolide resulted in 2.0-, 2.5-, and 5.4-fold increase in apoptotic events, respectively, compared to those in the DMSO-treated control. Caspase-3 activation, which serves as an indicator of apoptosis, was observed in BxPC-3 cells treated with 5-*epi*-sinuleptolide (Figure 3c). Due to the increasing number of cells showing activated Caspase-3, Caspase-3 activation was suggested to be involved in 5-*epi*-sinuleptolide-induced apoptosis. Taken together, these results suggest that the 5-*epi*-sinuleptolide-mediated cytotoxicity in BxPC-3 cells may be attributed to both the inhibition of cell proliferation and the induction of apoptosis.

### 2.3. 5-epi-Sinuleptolide Induced the G2/M Arrest by Regulating the Expression of the Mitosis-Regulating Factors

**Figure 4:**
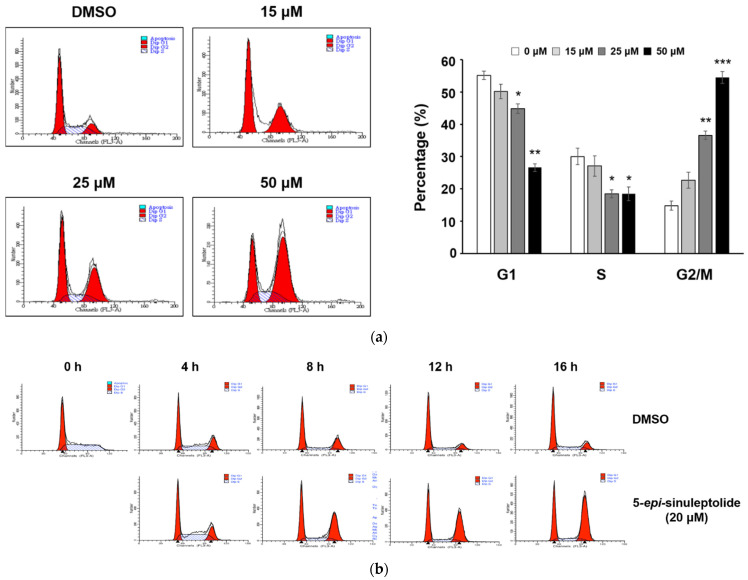

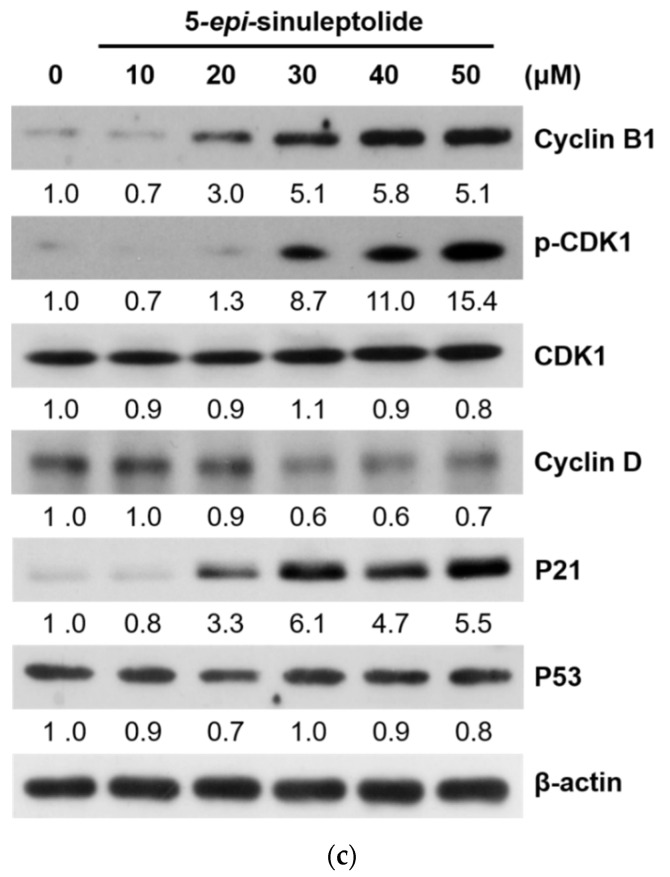
The aberrant cell survival of pancreatic cancer cells after 5-*epi*-sinuleptolide treatment is partially due to the inhibition of cell proliferation, especially G2/M arrest. Cell cycle analysis via flow cytometry using propidium iodide-stained BxPC-3 cells. Cells were treated for 24 h with 15, 25, and 50 μM 5-*epi*-sinuleptolide. Data shown are representative of three independent experiments. The percentages of cells in the G1, S, and G2/M phase at each dose are illustrated as a bar graph shown in the right-hand side. Data are expressed as the mean ± standard deviation from at least three independent experiments. * indicates *p* < 0.05 vs. DMSO-treated control group, ** indicates *p* < 0.01, and *** *p* < 0.001 (**a**). DMSO- and 5-*epi*-sinuleptolide-treated cells were released from a double-thymidine block, and cell cycle distribution was determined at the indicated time points. The cell cycle profile shown was obtained from one of three independent experiments (**b**). Representative Western blot bands showing the expression of proteins associated with G2/M progression; β-actin served as a loading control (**c**). The numbers under each blot represent values of corresponding band intensity relative to that of actin and untreated control.

Since the effect on apoptosis is not as prominent as proliferation inhibition, we considered that the decline in cell viability might have been due to the suppression of the cell cycle progression. We examined the effect of 5-*epi*-sinuleptolide on cell cycle progression, using flow cytometry analysis after staining the treated BxPC-3 cells with PI. The percentage of BxPC-3 cell population in G2/M phase increased from 14.76 ± 1.44% (DMSO control) to 36.63 ± 1.31% and 54.53 ± 1.88% after incubation with 25 and 50 µM of 5-*epi*-sinuleptolide, respectively. These results indicate that growth inhibitory effects of 5-*epi*-sinuleptolide involve cell cycle arrest at G2/M phase in a dose-dependent manner (Figure 4a). We further used a double-thymidine block to synchronize BxPC-3 cells at the G1 phase and monitored the cell cycle progression every 4 h. Cells treated with 5-*epi*-sinuleptolide accumulated at the G2/M phase without release even after 16 h (Figure 4b). These data suggest that 5-*epi*-sinuleptolide induced the G2/M arrest in BxPC-3 cells. To determine the mechanisms underlying the G2/M cell cycle arrest induced by 5-*epi*-sinuleptolide treatment, the expression levels of several G2/M progression-related proteins were assessed (Figure 4c). Cyclin-dependent kinase 1 (CDK1), the protein kinase that drives the mitotic state, and its cyclin partner cyclin B1 are essential for triggering mitotic entry and maintenance of the mitotic state in mammalian cells [19], whereas the inactivation of CDK1 and cyclin B1 destruction are required for exiting from mitosis [20]. Inefficient degradation of cyclin B1 results in constitutively active CDK1 and indefinite arrest in mitosis [21]. As shown in Figure 4c, treatment with 5-*epi*-sinuleptolide dose-dependently increased the expression of cyclin B1 and phosphorylation status (p) of CDK1. The sustained high cyclin B1–CDK1 activity might get cells stuck in the mitotic phase and cause cell cycle arrest. In addition, cyclin D is an important cell cycle regulator throughout the cell cycle, and its expression was suppressed via 5-*epi*-sinuleptolide treatment. P21, a transcriptional target of P53, is known to induce the S phase or G2/M arrest via the inhibition of CDKs [22]. Treatment with 5-*epi*-sinuleptolide resulted in the induction of p21; however, the consistent expression of p53 suggested that the cell cycle arrest mediated by 5-*epi*-sinuleptolide may be independent of p53.

### 2.4. 5-epi-Sinuleptolide Decreased the Invasion Ability of Pancreatic Cancer Cells and Suppressed the Protein Kinase B (AKT), Extracellular Signal-Regulated Kinase (ERK) 1/2, and Janus Kinase 2 (JAK2)/Signal Transducer and Activator of Transcription 3 (STAT3) Pathways

**Figure 5:**
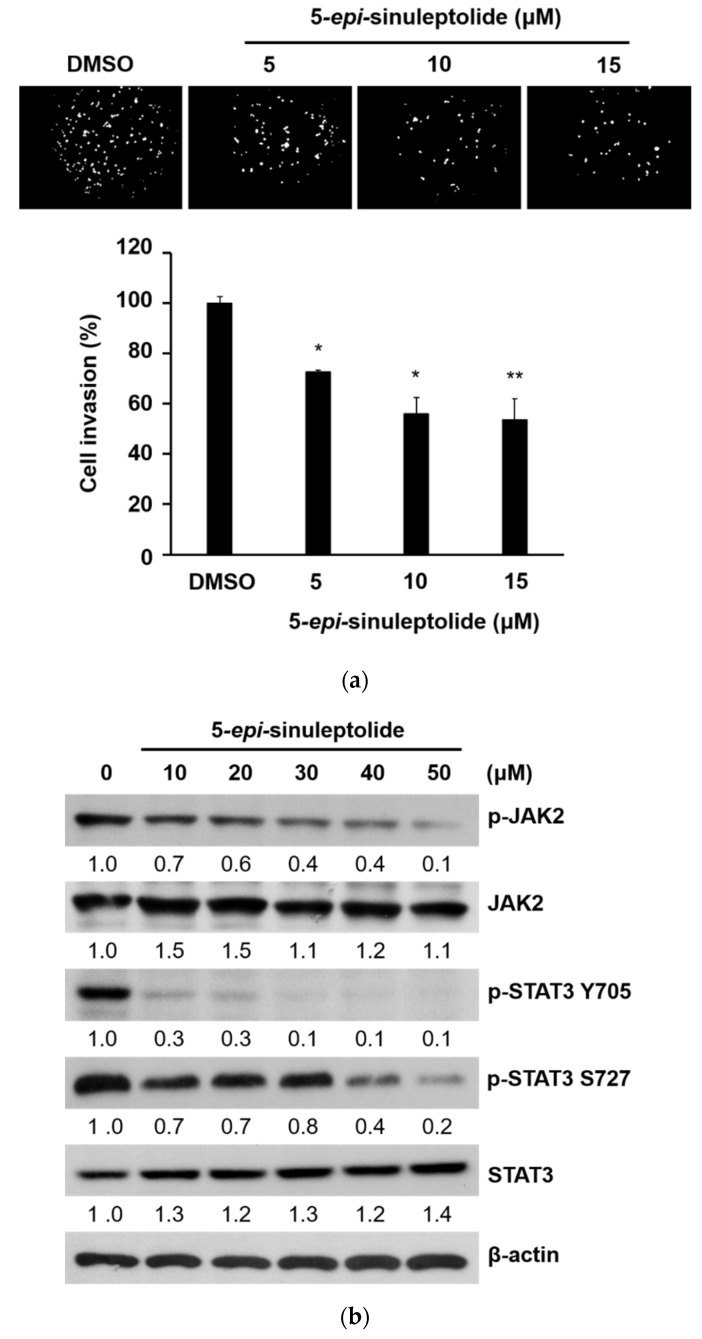

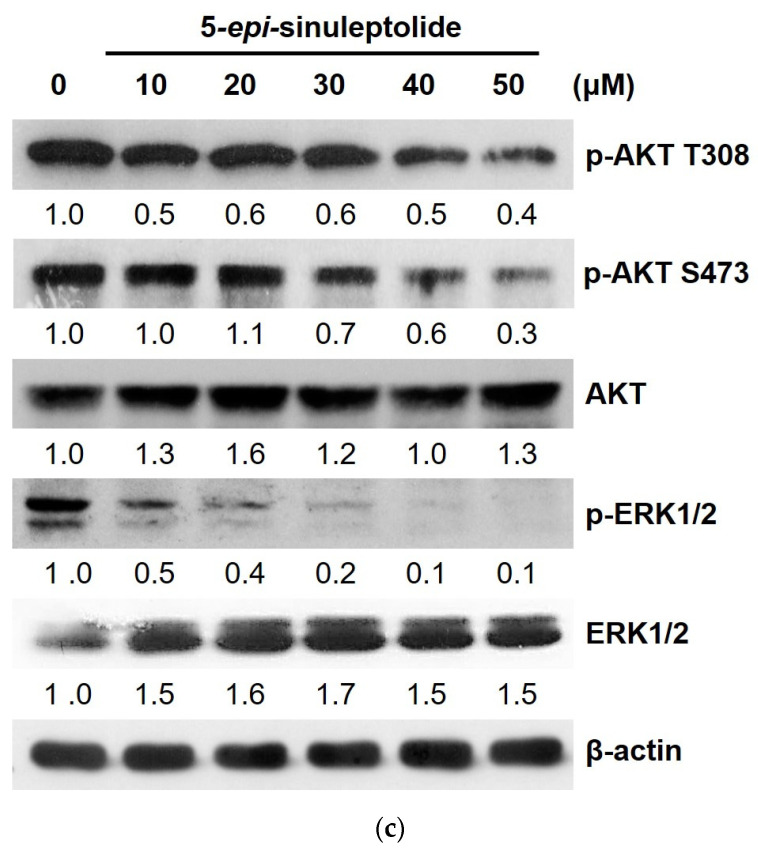
Cell invasion and JAK2/STAT3, AKT, ERK1/2 pathways are suppressed via 5-*epi*-sinuleptolide treatment. The invasiveness of cancer cells was examined via Matrigel invasion assays. PANC-1 cells were treated with 5*-epi*-sinuleptolide at the indicated concentrations for 24 h. The invading cells were stained with DAPI and evaluated via fluorescent microscopy. Bars represent the mean of triplicate samples; error bars represent standard deviation. Data are representative of three independent experiments. * *p* < 0.05 and ** *p* < 0.01 versus cells with DMSO treatment (**a**). Representative Western blot bands showing protein expression of the endogenous and phosphorylated JAK2, STAT3, AKT, and ERK1/2 levels in BxPC-3 cells treated with indicated concentrations of 5-*epi*-sinuleptolide. DAPI, 4′,6-diamidino-2-phenylindole; JAK2, Janus kinase 2; p-JAK2, phosphorylated JAK2; STAT3, signal transducer and activator of transcription 3; AKT, protein kinase B; and ERK1/2, extracellular signal-regulated kinase 1/2 (**b**,**c**). The numbers under each blot represent values of corresponding band intensity relative to that of actin and untreated control.

Local cancer cell invasion represents the initial step of metastasis that dramatically worsens prognosis and patient survival. Numerous attempts have been made to interfere with this early event and to eradicate metastasis at its initiation [23]. An invasion assay was performed to determine the effect of 5-*epi*-sinuleptolide treatment on the invasion of pancreatic cancer cells. The invasiveness of PANC-1 cells was significantly suppressed via 5-*epi*-sinuleptolide treatment (Figure 5a). This reduction was dose-dependent, with a 27%, 53%, and 69% decrease when cells were treated with 5, 10, and 15 μM of 5-*epi*-sinuleptolide, respectively. These results suggest that 5-*epi*-sinuleptolide may possess an anti-metastatic potential by inhibiting the invasion of pancreatic cancer cells. We further assessed the expression patterns of proteins involved in several classic pathways via western blotting to determine the underlying mechanism by which 5-*epi*-sinuleptolide exerted its actions. The JAK/STAT signaling pathway plays a multitude of important biological functions in cell growth, differentiation, survival, and metastasis in many human cancers [24,25]. The effects of 5-*epi*-sinuleptolide on the expression of these proteins were evaluated. The phosphorylation of JAK2 and STAT3 in BxPC-3 cells was markedly inhibited after 24 h of treatment with 5-*epi*-sinuleptolide (Figure 5b). To investigate the role of AKT and ERK in the proliferation and motility of pancreatic cancer cells, the activation status of AKT and ERK1/2 in BxPC-3 cells was also examined. The levels of phosphorylated AKT and ERK1/2 were effectively suppressed in BxPC-3 cells after treatment with 5-*epi*-sinuleptolide (Figure 5c). Collectively, these results suggest that 5-*epi*-sinuleptolide could inhibit the activities of key regulators for cancer progression including JAK2/STAT3, AKT, and ERK1/2, and suppress the invasiveness of malignant pancreatic cells.

## 3. Discussion

Pancreatic cancer is one of the deadliest of all types of cancer with an extremely poor prognosis [26]. Despite a growing number of targeted and molecular therapies offering hope for more patients with various cancers and have greatly improved their survival, treatment outcomes for pancreatic cancer have not changed considerably during the last three decades. Gemcitabine is a standard therapy for advanced pancreatic cancer; however, the median survival time for patients treated with single-agent gemcitabine has only ranged from 5.6 to 6.3 months [27]. The minor impact on the overall survival (OS) of patients with locally advanced or metastatic disease comprises the majority of cases [28]. In addition, gemcitabine-based chemotherapy is commonly confederated with severe side effects and drug resistance [29]. The current treatment for metastatic PDAC includes combination chemotherapy, such as FOLFIRINOX [30] or co-treatment with gemcitabine and nab-paclitaxel [31]. Although the combination regimens have prolonged the median OS to 8.5 months; these treatments create a considerable toxic burden. Many attempts have been made in the past decades to improve systemic therapies in pancreatic cancer, but they have either failed to advance efficacy or induce considerable toxic side effects [32,33,34]. Therefore, there is an unmet clinical demand for effective chemotherapy to manage patients with pancreatic cancer.

**Figure 6:**
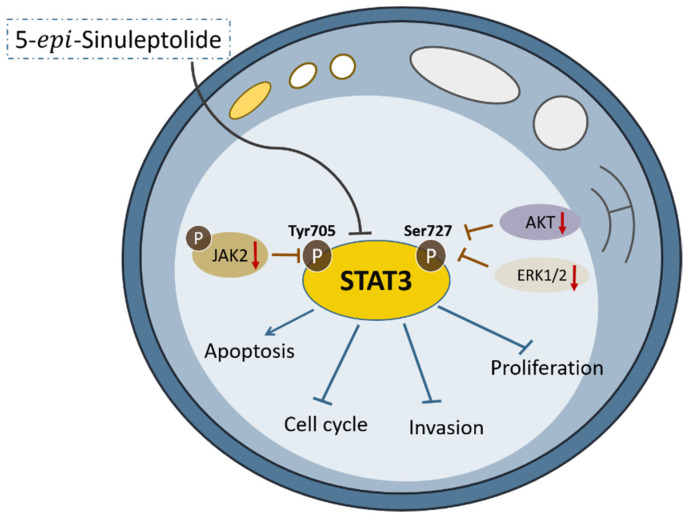
Schematic illustration of the mechanism of the cytotoxic effects of 5-*epi*-sinuleptolide on pancreatic cancer cells.

In the present study, we showed that 5-*epi*-sinuleptolide, a compound from the soft coral genus *Sinularia,* could inhibit pancreatic cancer cell proliferation, induce cell cycle arrest at the G2/M phase, trigger apoptosis, and suppress cell invasion to a great extent. We further examined the expression levels of G2/M transition-related proteins after exposure to different concentrations of 5-*epi*-sinuleptolide and found that the inactive CDK1/cyclin B1 complex may contribute to the failure of G2/M transition. In addition, the suppression of phosphorylation levels of JAK2/STAT3, AKT, and ERK1/2 may account for the diverse cytotoxic effects of 5-*epi*-sinuleptolide (Figure 6).

Cell cycle arrest is an active response to stresses that prevents cell proliferation and division in defective cells. S- and M-phases are the most crucial events that allow for the correct cell duplication without accumulating genetic errors, so the cell cycle arrest mostly occurs at the G1/S or G2/M transition [35]. Active CDK1 complexed to cyclin B1 is required for progression from G2 to M phases. When the CDK1/cyclin B1 complex is inactivated by P21, the cell cycle ceases at the G2 checkpoint [36]. P21 expression was remarkably increased after 5-*epi*-sinuleptolide treatment, whereas P53 expression remained unaltered (Figure 4c). P53 is considered an upstream regulator of P21; however, P53 mutations have been shown in 95% of the pancreatic cancer cell lines including PANC-1 and BxPC-3 used in this study [37]. P21 induction by 5-*epi*-sinuleptolide may be accomplished by P53-independent regulation [38].

Several recent studies have supported the crucial role of activated STAT3 in various cancers [39,40,41]. STAT3 activation is induced by phosphorylation on a critical tyrosine residue (Tyr705), and such phosphorylation can be catalyzed by various tyrosine kinases including epidermal growth factor receptor (EGFR), platelet-derived growth factor receptor (PDGFR), vascular endothelial growth factor receptor (VEGFR), and colony stimulating factor-1 (CSF-1) [42,43]. STAT3 can also be constitutively activated by upstream signaling components, including increased cytokine (interleukin 6 and interleukin 10) production and non-receptor tyrosine kinases (including JAKs and Src) [44]. In addition to tyrosine kinases, various serine kinases such as mitogen-activated protein kinase (MAPK) (p38 MAPK, ERK, and JNK), protein kinase C-delta, mechanistic target of rapamycin, and serine/threonine-protein kinase have been reported to phosphorylate STAT3 at serine position 727 (Ser727), which is required for the maximal transcriptional activity of STAT3 [45,46]. The STAT3 protein is phosphorylated and dimerized upon activation, leading to nuclear translocation of p-STAT3, with significant overexpression of several target genes downstream of STAT3 involved in a variety of biological processes [47,48], such as cell cycle regulation, evasion of apoptosis, invasion and migration, and angiogenesis.

STAT3 is constitutively activated in pancreatic cancer via phosphorylation of Tyr705, as found in human tumor specimens as well as in various pancreatic cancer cell lines [49,50]. An increasing number of studies have shown that STAT3 activation plays a pivotal role in the progression, metastasis, and drug resistance of pancreatic cancer [51,52]. Our present study showed that 5-*epi*-sinuleptolide effectively inhibited the phosphorylation of both tyrosine 705 and serine 727 sites of STAT3 and the consequent downstream cellular effects (inhibition of cell proliferation, induction of apoptosis, and suppression of invasiveness) in pancreatic cancer cells.

AKT has been shown to be an important effector of oncogenic Ras, which regulates cellular processes such as cell proliferation, differentiation, migration, apoptosis, and drug resistance [53]. A striking feature of pancreatic cancer is that mutationally activated K-ras is present in ∼90% of PDAC cases. As a key downstream target of the Ras family, AKT activation is a frequent event and correlates with the outcome in approximately 60% of pancreatic cancers [54]. Overexpression and activation of AKT has been associated with worse prognostic variables and outcome, as well as the apoptotic effect of chemotherapy [55,56]. Treatment with 5-*epi*-sinuleptolide induced a dose-dependent reduction in AKT phosphorylation at both threonine 308 and serine 473 sites, thereby inhibiting cell growth and inducing apoptosis.

The ERK pathway is involved in cellular proliferation, differentiation, and survival. The activated ERK pathway promotes cell proliferation and survival in pancreatic cancer cells; contrariwise, inhibition of the ERK pathway promotes apoptosis via caspase cascade activation [57]. Notably, the levels of phosphorylated ERK were remarkably decreased via 5-*epi*-sinuleptolide treatment, and the survival/apoptotic pathways were affected to induce growth arrest.

Taken together, our study demonstrates for the first time that 5-*epi*-sinuleptolide can markedly inhibit the growth and metastasis of PDAC cells. Mechanistically, we found that the AKT, ERK, and JAK2/STAT3 pathways account for the cytotoxic effects of 5-*epi*-sinuleptolide. These results imply that 5-*epi*-sinuleptolide may represent a promising therapeutic drug for patients with pancreatic cancer.

## 4. Materials and Methods

### 4.1. Reagents and Cell Culture

Sinuleptolide and 5-*epi*-sinuleptolide were isolated from *Sinularia leptoclados* and identified by Jyh-Horng Sheu, National Sun Yat-sen University, Kaohsiung, Taiwan. Figure 1 shows the chemical structures of sinuleptolide and 5-*epi*-sinuleptolide. Human pancreatic cancer cell lines (BxPC-3 and PANC-1) were purchased from the Bioresource Collection and Research Center in Taiwan. BxPC-3 was cultured in RPMI-1640, while PANC-1 was grown in DMEM medium (Gibco-Invitrogen) supplemented with 10% fetal bovine serum (FBS) and 1% antibiotics. The immortalized HPDE-E6E7 pancreatic duct epithelial cell line was kindly provided by Dr. Yan-Shen Shan and cultured in keratinocyte serum-free (KSF) medium supplemented by epidermal growth factor and bovine pituitary extract (Life Technologies, Inc., Grand Island, NY, USA).

### 4.2. Cell Viability Assay

The MTT (3-[4,5-Dimethylthiazol-2-yl]-2,5-Diphenyltetrazolium bromide) assay was performed to determine the cytotoxicity of 5-*epi*-Sinuleptolide as described previously [58]. Briefly, cells were plated in 96-well plates at a density of 5 × 10^3^ cells per well and incubated overnight at 37 °C. The cells were then treated with 10, 20, 30, 40, and 50 μM 5-*epi*-sinuleptolide for 24 h. DMSO was used as a vehicle control. After treatment, the medium was replaced with 150 μL of medium containing 10% MTT solution (Sigma-Aldrich, St. Louis, MO, USA). After 1 h of incubation at 37 °C, the purple formazan crystals were dissolved in DMSO and the absorbance was recorded on a microplate reader at a wavelength of 595 nm. The cell viability was calculated by normalizing their absorbance to that of the corresponding control sample and represented as the mean ± standard deviation of six independent experiments performed in triplicate.

### 4.3. Proliferation Assay

Briefly, 5000 cells/100 μL/well cultured in 96-well plates were treated with 10, 20, 30, 40, and 50 μM 5-*epi*-sinuleptolide for 24 h. After treatment, cells in each well were labeled with BrdU for 4 h at 37 °C using the Cell Proliferation enzyme-linked immunosorbent assay (ELISA), BrdU (colorimetric) Kit (Roche Applied Science, Indianapolis, IN, USA) according to the manufacturer’s instructions. After fixation, the cells were incubated with anti-BrdU-peroxidase antibody (1:100) for 30 min at room temperature. Following the substrate reaction, the absorbance of the reaction product was measured using an ELISA reader at 450 nm wavelength.

### 4.4. Annexin V/Propidium Iodide (PI) Assay

Pancreatic cancer cell death induced by 5-*epi*-sinuleptolide was examined by using the Dead Cell Apoptosis Kit with Annexin V-Alexa Fluor™ 488 & PI (Invitrogen, Carlsbad, CA, USA) according to the manufacturer’s instructions. Briefly, after 24 h of treatment with 5, 25, and 50 μM of 5-*epi*-sinuleptolide or DMSO (control), the cells were stained with Annexin V and PI (5 μg/mL) and then analyzed via flow cytometry (BD Biosciences, Palo Alto, CA, USA).

### 4.5. Caspase-3 Activation Assay

Caspase-3 activity was measured using the FITC Active Caspase-3 Apoptosis Kit (BD Biosciences) according to the manufacturer’s instructions. Briefly, pancreatic cancer cells were seeded at a density of 1 × 10^6^ cells per P10 dish and were cultured overnight. After 5, 25, and 50 μM of 5-*epi*-sinuleptolide or DMSO treatment for 24 h, harvested cells were fixed and permeabilized by Cytofix/Cytoperm solution at 4 °C for 20 min. Cleaved Caspase-3 labeling was performed by incubating the cells with FITC-conjugated anti-active caspase-3 antibody for 30 min at room temperature. Caspase-3 activity was measured and analyzed via flow cytometry and by using the WinMDI 2.9 software (BD Biosciences).

### 4.6. Cell Cycle Analysis

Approximately 70% confluent BxPC-3 cells were treated with 15, 25, and 50 μM of 5-*epi*-sinuleptolid for 24 h. Before staining with PI (Sigma-Aldrich), cells were fixed overnight with 70% ethanol at 4 °C. The cells were washed twice with ice-cold PBS (1×), resuspended in RNase A (50 μg/mL), PI (40 μg/mL), and PBS in a total volume of 500 μL at 37 °C for 30 min. The stained cells were further analyzed via flow cytometry and the percentage of cells in each phase of the cell cycle was determined using Modfit (Verity Software House Inc., Topsham, ME, USA). For S-phase synchronization by double thymidine block, BxPC-3 cells were grown in the presence of thymidine (2 mM) for 18 h, transferred to thymidine-free medium for 6–8 h, and finally cultured again in 2 mM thymidine-containing medium for 12 h. Cells were then washed twice with PBS followed by the addition of regular culture media containing DMSO or 20 μM of 5-*epi*-sinuleptolid. Cells were collected every four hours for cell cycle analysis.

### 4.7. Invasion Assay

Matrigel (BD Bioscience, Bedford, MA, U.S.A.) was added to Transwell inserts at a concentration of 1 mg/mL and consolidated at 37 °C overnight. Subsequently, 2 × 10^4^ cells were mixed with serum-free medium containing DMSO or 5, 10, and 15 μM of 5-*epi*-sinuleptolide and were placed in the upper chamber and were allowed to migrate toward the bottom chamber containing culture medium with 10% FBS for 24 h. The invasive cells that had reached the lower side of the membranes were stained with 5 μg/mL 4′,6-diamidino-2-phenylindole (DAPI). The number of invading cells was counted in five random fields (40×) via fluorescence microscopy.

### 4.8. Western Blotting

A total of 1 × 10^6^ cells were treated with 10, 20, 30, 40, and 50 μM of 5-*epi*-sinuleptolide or DMSO (control) for 24 h. Treated cells were washed and lysed in radioimmunoprecipitation acid (RIPA) lysis buffer (Cell Signaling Technology, Beverly, MA, USA) containing 1% protease inhibitor for 5 min on ice and then subjected to sonication for 20 s. The total protein was determined using Bio-Rad protein assay solution. For immunoblotting, 20 μg protein samples were processed, separated on 7.5%–12.5% SDS-PAGE gels, and transferred onto the PVDF membrane (Millipore, Bedford, MA, USA). After blocking in 5% skim milk for 1 h at room temperature, the blots were hybridized with primary antibodies against Cyclin B1 (1:1000, sc-245, Santa Cruz, CA, USA), Cyclin D1 (1:1000, sc-8396), P21 (1:1000, sc-6246), P53 (1:1000, sc-126), β-actin (1:1000, sc-47778), p-CDK1/CDK1 (1:500, #9111/#9116, Cell Signaling Technology), p-JAK2/JAK2 (1:500, #3230/#8082), p-STAT3(Y705)/p-STAT3(S727)/STAT3 (1:500, #9131/#9134/#9139), p-AKT(T308)/p-AKT(S473)/AKT (1:500, #13038/#4060/#4691), p-ERK1/2 and ERK1/2 (1:500, #4370/#4695, Cell Signaling Technology) and incubated with a goat anti-rabbit or anti-mouse secondary antibody (1:10000, ab205718/ab6708, Abcam), respectively, as described [58]. Finally, the protein of interest was detected using ECL Western Blotting Detection System (GE Healthcare, Buckinghamshire, UK), and Image J software (National Institutes of Health, Bethesda, MD, USA) was employed for analysis. Western blot assay was carried out at least three times.

### 4.9. Statistical Analysis

All data are presented as the mean ± standard deviation. The Student’s two-tailed *t*-test was used to test the significance of differences between the treatment and control values. Differences were considered significant at *p* < 0.05.

## Sample Availability

Not available.
